# Body composition as a complementary tool for detection of metabolic syndrome 6 years postpartum: a St. Carlos Cohort follow-up

**DOI:** 10.3389/fnut.2025.1689658

**Published:** 2025-10-29

**Authors:** Bricia López-Plaza, Angélica Larrad-Sainz, Johanna Valerio, Rocío Martín O’Connor, Laura del Valle, Ana M. Ramos-Levi, Ana Barabash, Clara Marcuello, Inés Jiménez-Varas, Miguel A. Rubio-Herrera, Pilar Matía-Martín, Alfonso L. Calle-Pascual

**Affiliations:** ^1^Departamento de Endocrinología y Nutrición, Hospital Clínico San Carlos, Instituto de Investigación Sanitaria San Carlos (IdISSC), Madrid, Spain; ^2^Departamento de Medicina II, Facultad de Medicina, Universidad Complutense de Madrid, Madrid, Spain; ^3^Centro de Investigación Biomédica en Red de Diabetes y Enfermedades Metabólicas Asociadas (CIBERDEM), Madrid, Spain

**Keywords:** gestational diabetes mellitus, body composition, metabolic syndrome, postpartum period, prevalance

## Abstract

**Background and aims:**

Gestational diabetes mellitus (GDM) is a prevalent pregnancy complication associated with long-term cardiometabolic risk, including metabolic syndrome (MetS). This study aimed to assess differences in body composition and metabolic health 6 years postpartum based on prior GDM diagnosis and to identify body composition cut-off values predictive of MetS.

**Methods:**

This cross-sectional analysis included 604 women from the prospective St. Carlos Cohort in Spain, who had no subsequent pregnancies and complete body composition data 6 years postpartum. Body composition was assessed using bioelectrical impedance analysis (BIA), and MetS was diagnosed per harmonized criteria. Statistical analyses included ROC curves to establish diagnostic accuracy and optimal cut-off points.

**Results:**

Women with prior GDM had a twofold increased risk of developing MetS (26.6 vs. 14.6%). However, waist circumference or elevated BMI and waist-to-height ratio were not significantly different between groups. ROC analysis identified that body composition parameters, particularly fat mass (FM), visceral fat, and FM/Fat Free Mass ratio, as having high predictive value for MetS, regardless of GDM history (AUC ≥ 0.8). Women with MetS showed significantly higher FM and lower relative muscle mass and function. Diagnostic models showed high negative predictive values (≥90%) for most body composition parameters making them effective for excluding MetS.

**Conclusion:**

GDM is a significant predictor of MetS. However, body composition, especially increased adiposity and reduced relative muscle mass, provides valuable clinical insights beyond traditional anthropometric measures in postpartum women. The proposed cut-off values for body composition parameters may serve as effective, non-invasive tools for early MetS detection in postpartum care.

## 1 Introduction

Gestational diabetes mellitus (GDM) is characterized by hyperglycemia first recognized during the second or third trimester of pregnancy, in cases where overt diabetes is not clearly present (1, 2). According to the most recent report by the International Diabetes Federation (IDF), approximately 14% of pregnancies worldwide are affected by GDM when applying the criteria established by the International Association of Diabetes and Pregnancy Study Groups (IADPSG) (3). In Europe, the prevalence is slightly lower, at 7.8%, while in Spain is around 13.9%, depending on the population studied and diagnostic criteria used (3, 4).

Risk factors for GDM include overweight or obesity, advanced maternal age, excessive gestational weight gain, ethnicity, family history of insulin resistance or diabetes, among others (5). Although GDM usually resolves after delivery, women diagnosed with GDM have a significantly increased risk of developing type 2 diabetes mellitus and cardiovascular disease in the long-term follow-up (6, 7). Furthermore, women with a history of GDM also have a higher risk (8) and prevalence of metabolic syndrome (MetS) (25.3%) compared to those without GMD history (6.6%) (9). Women diagnosed with GDM often present long-term alterations in body composition after childbirth (10, 11). Normoglycemic women who experience excessive gestational weight gain also entail an increased risk of postpartum weight retention and potentially unfavorable changes in body composition (12, 13). These findings suggest that pregnancy itself may trigger persistent metabolic alterations, which could contribute to the later development of cardiometabolic disease (14, 15). Consequently, body composition may serve as a useful prognostic marker for MetS in women with a history of pregnancy.

The St. Carlos Cohort is a prospective population-based clinical study established at the Hospital Clínico San Carlos in Madrid, Spain. It was designed to analyze long-term outcomes in women diagnosed with GDM and to identify modifiable risk factors contributing to the development of subsequent metabolic disorders. The cohort integrates data from three public funded national studies and includes follow-up of over 2,500 women for more than 10 years (16). The St. Carlos Cohort represents a substantial contribution to the understanding and prevention of long-term metabolic complications in women with prior GDM, offering valuable insights for the development of early, targeted, and personalized intervention strategies (17).

In this setting, the present study was conducted with two main objectives: first, to evaluate differences in maternal body composition and metabolic health 6 years postpartum according to previous GDM diagnosis; and second, to establish, for the first time, cut-off points for body composition parameters associated with the risk of MetS.

## 2 Materials and methods

The St. Carlos Cohort comprises three consecutives prospective, single-center, interventional clinical trials all registered at https://www.isrctn.com/ under the identifiers ISRCTN84389045, ISRCTN13389832, and ISRCTN16896947. The data used in the present analysis were collected in an identical manner across all three studies, thereby enabling the integration of the study variables.

### 2.1 Participants and selection criteria

**Figure 1:**
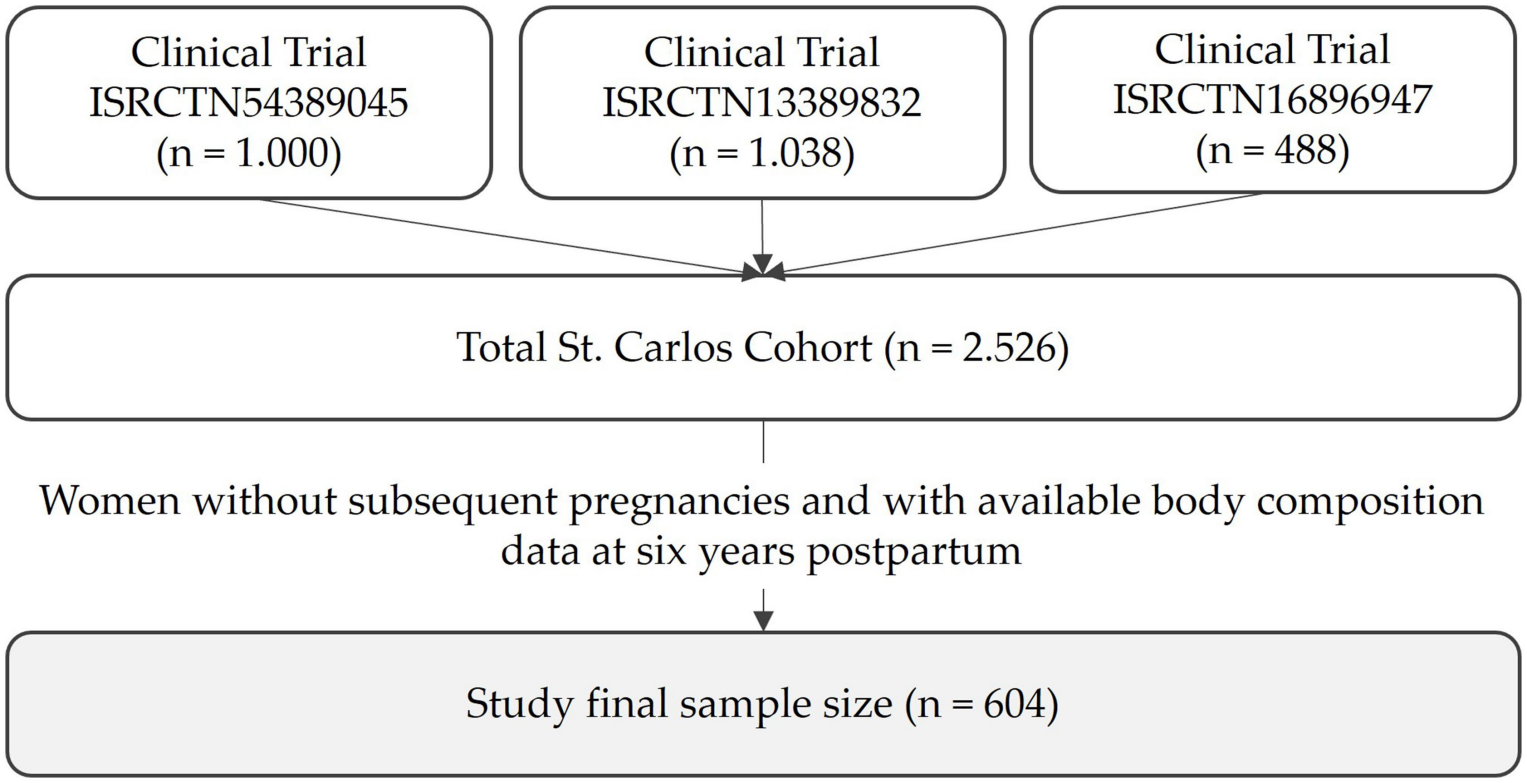
Constitution of the St. Carlos Cohort and final size of the study.

At baseline, a total of 2,529 normoglycemic pregnant women were enrolled in the St. Carlos Cohort at approximately the 12th gestational week (GW). All participants were assessed and closely monitored by both the Department of Obstetrics and the Department of Nutrition at the Hospital Clínico San Carlos in Madrid, Spain. Of these, 2,228 participants completed antenatal follow-up all the way through to delivery and were evaluated at the end of pregnancy. Longitudinal follow-up during gestation and the postpartum period was conducted from 2015 to 2018. A total of 1,403 women completed a face-to-face visit at 6 years postpartum and were subsequently included in the postnatal phase of the study. For the present cross-sectional analysis from the prospective St. Carlos Cohort, only women without subsequent pregnancies and with available data on body composition at 6 years postpartum were considered, resulting in a final sample of 604 participants (Figure 1). Women with subsequent pregnancies during the follow-up period were excluded in order to avoid the potential confounding effects of additional gestational exposures on metabolic outcomes.

### 2.2 Ethics statement

The three studies were approved by the Ethics Committee of Hospital Clínico San Carlos under the codes CI 13/296-E, CI 16/442-E, and CI 16/316.^1^ All procedures were conducted in accordance with the Ethical Standards of the Institutional Research Committee and the principles outlined in the Declaration of Helsinki for biomedical research involving human participants (18). All researchers involved known and followed the ICH Harmonized Tripartite Guidelines for Good Clinical Practice (19).

All study data were processed by members of the research team in a database specifically created for this study and dissociated from any data that could identify the patient. The processing of personal data will follow the Spanish Organic Law (Ley Orgánica) 3/2018, of December 5, and the General Data Protection Regulation of the European Union (EU) 2016/679 of April 27, 2016.

### 2.3 Timeline

**Figure 2:**
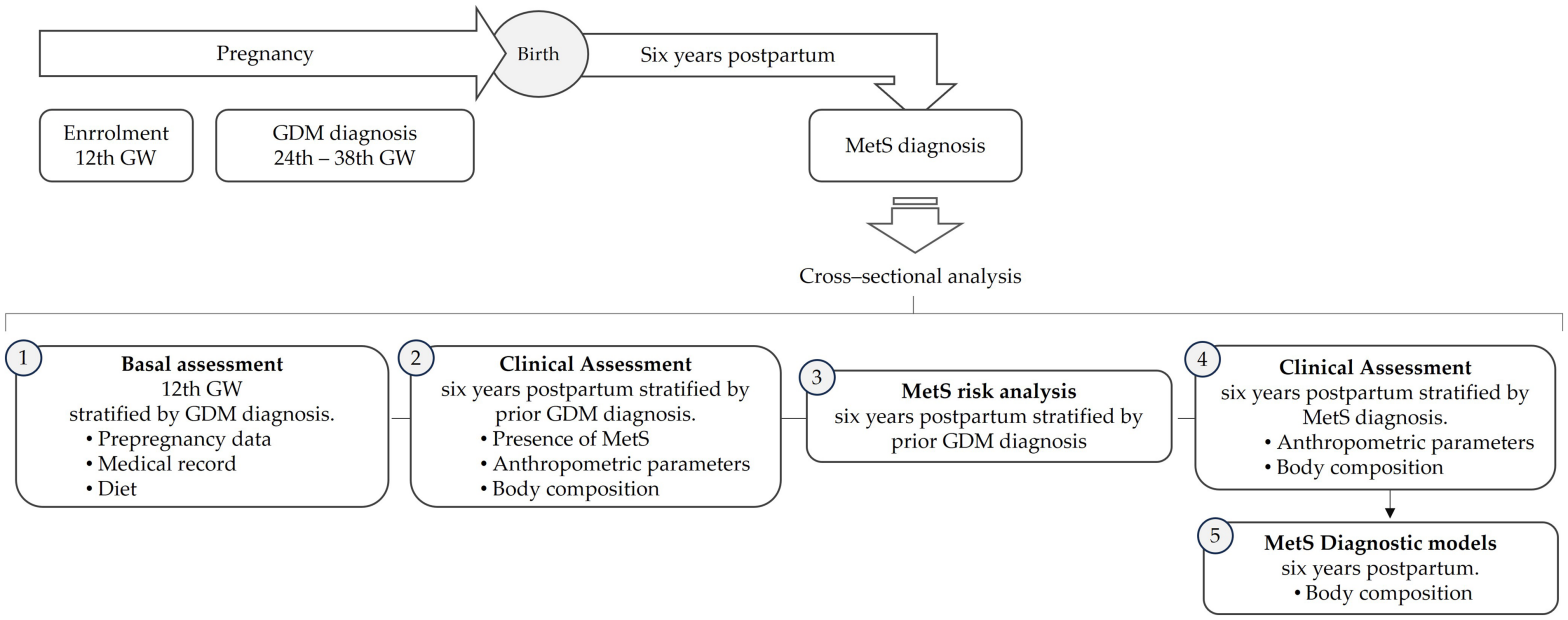
Flowchart of the analysis of the study.

The prospective St. Carlos Cohort and the present cross-sectional analysis are represented in Figure 2. Baseline characteristics were initially evaluated at the 12th GW and stratified by GDM during pregnancy. A follow-up assessment was conducted 6 years postpartum, examining the presence of MetS, anthropometric parameters, and body composition, also stratified by prior GDM diagnosis. During this phase, the risk of developing MetS 6 years after delivery was analyzed in relation to the previous diagnosis of GDM. Finally, body composition was evaluated based on the presence of MetS after 6 years. In this stage, diagnostic models for MetS were also developed and their performance was evaluated.

### 2.4 Definition of gestational diabetes mellitus

GDM was diagnosed according to the criteria established by the International Association of the IADPSG (20, 21). Screening was performed between 24 and 28 GW using a single-step 2-h 75-g oral glucose tolerance test (OGTT). Plasma glucose levels were measured at fasting, 1 h, and 2 h post-glucose load. Diagnosis of GDM was made when one or more of the following thresholds were met: fasting plasma glucose (FPG) ≥ 92 mg/dL, 1-h plasma glucose ≥ 180 mg/dL, or 2-hour plasma glucose ≥ 153 mg/dL. Additionally, overt diabetes in pregnancy was diagnosed if any of the following criteria were met: FPG ≥ 126 mg/dL, glycated hemoglobin (HbA1c) ≥ 6.5%, or random plasma glucose ≥ 200 mg/dL, confirmed on a subsequent occasion.

### 2.5 Definition of metabolic syndrome

MetS was diagnosed according to the harmonized criteria proposed by the Joint Interim Statement (22). Participants were classified as having MetS if they met at least three of the following five criteria: waist circumference (WC) > 89.5 cm (women), triglycerides ≥ 150 mg/dL, HDL cholesterol < 50 mg/dL (women), blood pressure ≥ 130/85 mmHg, and FPG ≥ 100 mg/dL.

For the present study, WC thresholds were based on specific cut-off values previously established for the Spanish population (23). WC was not considered a mandatory criterion for the diagnosis of MetS.

### 2.6 Anthropometric parameters

Anthropometric variables were assessed using standardized procedures in accordance with the international guidelines established by the World Health Organization (WHO) (24). Body weight was measured in the morning after a 12-hour overnight fast, following evacuation, using a calibrated digital clinical scale (capacity: 0–150 kg). Participants were barefoot and wore light clothing during measurement. Stature was measured to the nearest millimeter using a wall-mounted stadiometer (Seca 220®, Seca GmbH & Co. KG, Hamburg, Germany) with a measuring range of 80–200 cm. Body mass index (BMI) was calculated using the standard formula: weight (kg) divided by height squared (m^2^). WC was measured in centimeters with a non-elastic anthropometric tape, following the International Society for the Advancement of Kinanthropometry (ISAK) guidelines (25). WC was measured at the narrowest point of the torso when identifiable; otherwise, the measurement was taken midway between the lower margin of the last palpable rib and the top of the iliac crest. The waist-to-height ratio (WHtR) was subsequently calculated as WC (cm) divided by height (cm), and a WHtR ≥ 0.5 was considered indicative of increased cardiometabolic risk (26, 27).

### 2.7 Body composition

Body composition was assessed using a multifrequency medical body composition analyzer (mBCA 515®, Seca GmbH & Co. KG, Hamburg, Germany), which operates with eight electrodes: two pairs in contact with the hands and two pairs with the feet. The procedure followed the recommendations of the European Society for Clinical Nutrition and Metabolism (28, 29).

To minimize variability and ensure measurement reliability, the following standardized conditions were maintained prior to analysis: participants remained in a relaxed state, standing upright with bare feet and minimal clothing, limbs abducted at approximately 45°, and free from metallic accessories (e.g., earrings, necklaces, bracelets). Measurements were conducted in a thermoneutral environment, following a fasting period of at least 2 h and abstention from alcohol, coffee, caffeinated beverages, and chocolate for 24 h. Women were encouraged to avoid strenuous physical activity during the previous 24 h.

Bioelectrical impedance (BIA) was measured using a 100 μA current across a frequency range of 1–1,000 kHz. Raw values for resistance (R), reactance (Xc), and phase angle (PhA) were obtained at a frequency of 50 kHz. Impedance measurements at 5 and 50 kHz were used to develop the predictive equations (30). The PhA/BMI ratio was also calculated.

Body composition assessment submitted several key parameters. Total body water (TBW) and extracellular water (ECW) were quantified in liters, and their relative distribution was expressed as the ECW/TBW ratio (%). Fat mass (FM) was assessed in both absolute terms (kg) and as a percentage of total body weight (%). Fat-free mass (FFM) was measured in kilograms. Visceral adipose tissue was also estimated and reported in liters. To normalize for body size, FM and FFM indices were calculated by dividing each value by height squared (kg/m^2^) (31). Additionally, the FM to FFM ratio (FM/FFM) was determined to provide a further indicator of body composition balance (32).

Skeletal muscle mass (SMM) was estimated using the Janssen equation (33), and appendicular skeletal muscle mass (ASM) was calculated using the Sergi formula (34). Both were normalized by height squared to obtain the skeletal muscle mass index (SMMI) and the appendicular skeletal muscle mass index (ASMI), respectively (35). Relative SMM was also expressed as a percentage of total body weight (SMM/weight × 100) (33), and an additional index was calculated as SMM adjusted for BMI (SMM/BMI) (36).

### 2.8 Assessment of grip strength

Hand grip strength (HGS) was evaluated using a digital dynamometer (Jamar Plus Digital®, Performance Health International Ltd., Nottinghamshire, UK), a device with demonstrated high reliability and validity for assessing muscular strength (37). Measurements were performed on the dominant hand to assess upper body muscle function. The device features an adjustable handle with five grip positions (ranging from 35 to 87 mm), a digital scale calibrated in kilograms, and an isometric grip force range from 0 to 90 kg.

To ensure accuracy and reproducibility, HGS was assessed following standardized procedures based on established normative data for adults (38). Prior to testing, participants rested for at least 5 min in a seated position. Any jewelry or accessories that could interfere with grip performance were removed. The dynamometer handle was adjusted according to individual hand size to optimize grip alignment.

Participants were seated with the shoulder adducted and in a neutral rotation, the elbow flexed at 90°, and the wrist positioned between 0° and 30° of dorsiflexion and between 0° and 15° of ulnar deviation. The dynamometer was held in the dominant hand, with the handle aligned parallel to the fingers.

Subjects were instructed to exert maximal isometric force by squeezing the handle as hard as possible for 5 s after a verbal order. Three consecutive trials were conducted with 30-s rest intervals between attempts. The highest of three measurements was recorded as the final HGS value. In addition, the hand grip strength to body mass index ratio (HGS/BMI) was calculated to normalize strength relative to body size (39).

### 2.9 Dietary assessment

Adherence to the Mediterranean dietary pattern was evaluated using the Mediterranean Diet Adherence Screener (MEDAS) at two different time points. During pregnancy, a modified version of the MEDAS was performed (40). This version excluded items related to alcohol and fruit juice consumption, since these are not recommended during gestation. The adapted questionnaire consisted of 12 items, with a total score ranging from 0 to 12 points. Higher scores denoted greater adherence to the Mediterranean dietary pattern.

Assessment at the 6-year follow-up was conducted using the established 14-item MEDAS version (41). This validated tool assesses adherence based on the consumption of foods which are characteristic of the Mediterranean diet. These include beneficial components, such as vegetables, fruits, legumes, nuts, whole grains, fish, and olive oil, as well as items evaluating the intake of less recommended foods, such as red and processed meats, sugar-sweetened beverages, and commercial pastries. Each item is scored dichotomously (0 or 1) based on predefined consumption thresholds, resulting in a total score ranging from 0 to 14 points in the standard version. A score of 9 or higher was considered indicative of high adherence to the Mediterranean dietary pattern.

### 2.10 Physical activity

Physical activity was measured by the short version of the International Physical Activity Questionnaire (IPAQ) (42). This questionnaire consists of seven questions that explore physical activity patterns over the previous seven days and is divided into two main sections. The first section collects information on the type, frequency, and duration of activities performed in four areas: occupational, domestic, transportation, and leisure-time activities. Responses are recorded in terms of frequency (days per week) and duration (minutes per day). The second section assesses sedentary behavior by asking women to report the amount of time spent sitting on a typical weekday (hours per day). Once the questionnaire is completed, a total physical activity score is calculated, integrating the duration and frequency of reported activities, presenting both quantitative and qualitative information. The quantitative outcome is expressed as total energy metabolism equivalents (MET-minutes/week), whereas qualitative classification categorizes the physical activity as low, moderate, or high intensity.

For quantitative estimation, standardized MET values are applied to each activity category: 8 METs for vigorous activity, 4 METs for moderate activity, and 3.3 METs for walking. The total MET-minutes per week was calculated using the following formula:


$$\begin{array}{l} \mathrm{Total}\;\mathrm{MET}-\mathrm{minutes}/\mathrm{week}=\mathrm{MET}\;\mathrm{value}\times \mathrm{minutes}/\mathrm{day}\times \\ \mathrm{days}/\mathrm{week} \end{array}$$

### 2.11 Biochemical parameters

Blood samples were collected in the morning (between 08:00 and 09:00 h) following an overnight fast. Trained personnel at the Extraction Unit of Hospital Clínico San Carlos performed the venipuncture. Fasting blood samples were extracted into vacuum tubes and subsequently centrifuged at 1500×*g* for 10 min to obtain serum for biochemical analyses. Biochemical parameters were analyzed using standardized protocols specific to each assay. All determinations were performed by highly trained personnel from the Clinical Analysis Service, ensuring methodological accuracy and analytical reliability.

FPG was measured using the glucose oxidase method. Serum insulin concentrations were determined by chemiluminescent immunoassay on an IMMULITE 2000 Xpi system (Siemens Healthcare Diagnostics Inc., Tarrytown, NY, USA), with inter-assay coefficients of variation (CVs) of 6.3% at 11 μIU/mL and 5.91% at 21 μIU/mL. Insulin resistance was estimated using the homeostatic model assessment (HOMA) calculated as:


[glucose(mg/dL)×insulin(μIU/mL)]/405.

FPG and glycated hemoglobin (HbA1c) were standardized according to the International Federation of Clinical Chemistry and Laboratory Medicine (IFCC) using ion-exchange high-performance liquid chromatography (HPLC) with gradient elution on a Tosoh G8 analyzer (Tosoh Corporation, Minato-ku, Tokyo, Japan). Inter-assay imprecision for HbA1c at a concentration of 5.1% showed a standard deviation (SD) of 0.06 and a CV of 1.23%. At a concentration of 10.39%, the SD was 0.11 and the CV was 1.04%.

Total cholesterol was measured using the enzymatic colorimetric cholesterol oxidase-phenol aminophenazone (CHOD-PAP) method. Serum high-density lipoprotein cholesterol (HDL) concentration was determined by enzymatic immunoinhibition on an Olympus AU5800 analyzer (Beckman Coulter, Brea, CA, USA). Low-density lipoprotein cholesterol (LDL) was estimated using the Friedewald equation. Serum triglycerides were determined using a colorimetric enzymatic method based on glycerol phosphate oxidase-phenol aminophenazone (GPO-PAP).

Apolipoprotein B and high-sensitivity C-reactive protein (hsCRP) concentrations were measured using the Dimension Vista system (Siemens Healthcare Diagnostics, Munich, Germany), employing immunonephelometry and nephelometry, respectively.

Serum levels of aspartate aminotransferase (AST), alanine aminotransferase (ALT), gamma-glutamyl transferase (GGT), and alkaline phosphatase were measured using direct kinetic methods on an Olympus AU5800 analyzer (Beckman Coulter, Inc., Brea, CA, USA). Thyroid-stimulating hormone (TSH) levels were measured using a third-generation sandwich chemiluminescent immunoassay with magnetic particles and human TSH mouse monoclonal antibodies on a DXI-800® analyzer (Beckman Coulter, Inc., Brea, CA, USA). Free thyroxine (FT4) levels were determined using a two-step competitive chemiluminescent immunoassay with paramagnetic particles on the same analyzer.

All analytical procedures were subject to monthly external quality control through the Spanish Society of Clinical Chemistry (SEQC).

### 2.12 Statistical analysis

Continuous variables were expressed as mean and SD, whereas categorical variables were presented as absolute frequencies and percentages. Outliers were defined as values exceeding ±3 SD from the mean. The distribution of continuous variables was assessed using the Kolmogorov–Smirnov test, and homogeneity of variances was evaluated with Levene’s test. Depending on data distribution, comparisons between groups were conducted using either parametric tests (Student’s *t*-test) or non-parametric tests (Mann–Whitney *U* test). Categorical variables were analyzed using the chi-squared (*χ*^2^) test.

Odds ratios (ORs) with 95% confidence intervals (CIs) were calculated to evaluate the association between previous GDM diagnosis and the MetS risk at 6 years postpartum.

The receiver operating characteristic (ROC) curve and corresponding area under the curve (AUC) were used to determine the discriminatory capacity of body composition parameters in identifying MetS. AUC values ≥ 0.90 were classified as indicating excellent discrimination; values between 0.80 and 0.89 were considered very good discrimination; 0.70–0.79 reflected good discrimination; 0.60–0.69 indicated fair discrimination; and 0.50–0.59 were interpreted as poor discrimination. An AUC of 0.50 denotes no discriminative ability, equivalent to random classification. When a result fell below the line of no-discrimination, the method’s predictions were mirrored moving the result above the diagonal line. These values were identified with a prime symbol (‘). In such cases, values above the cut-off point indicated a lower risk of MetS. The Youden Index was applied to establish optimal cut-off points, maximizing both sensitivity and specificity. To assess the adequacy of the sample size, it has been performed a *post-hoc* power analysis based on two-tailed *Z* test for two independent proportions based on the observed prevalence of MetS in GDM and NGT groups.

All statistical tests were two-tailed, and a *p*-value < 0.05 was considered statistically significant. Analyses were performed using the Statistical Package for the Social Sciences (SPSS), version 25.0 for Windows (IBM Corp., Armonk, NY, USA) and the *post-hoc* statistical power analyses in G*Power Program version 3.1.9.7 for Windows (Düsseldorf University, Düsseldorf, Germany).

## 3 Results

Baseline results at 12th GW according to the diagnosis of GDM are shown in Table 1. A total of 604 women were selected from the 2,529 participants in the St. Carlos Cohort based on the availability of body composition data and the absence of subsequent pregnancies after a the 6-year postpartum follow-up. Among these women, 20.5% developed GDM during pregnancy. Advanced maternal age, higher prepregnancy weight and elevated BMI determined the development of GDM throughout their pregnancy. Specifically, an elevated prepregnancy BMI (≥25 kg/m^2^) raised the risk of developing GDM (OR = 1.784 [1.316–2.420]). An excess weight at the beginning of pregnancy was associated with a lower weight gain in women who later developed GDM. Additionally, individual constituents of MetS such as SBP or fasting blood glucose were also higher in women who developed GDM during pregnancy, although mean values for both parameters remained within normal range.

**Table 1:** Baseline characteristics at 12th GW stratified by gestational diabetes mellitus diagnosis (Mean ± SD).

			NGT (*n* = 480)	GDM (*n* = 124)	*P*-value
Age		(years)	34.17 ± 4.95	35.4 ± 5.04	0.014
Prepregnancy body weight		(kg)	60.57 ± 9.84	63.54 ± 12.54	0.015
Prepregnancy BMI		(kg/m^2^)	22.99 ± 3.49	24.36 ± 4.42	0.002
Prepregnancy BMI ≥ 25 kg/m^2^		(%)	109 (22.9)	42 (33.9)	0.012
Weight gain at	24–28 GW	(kg)	7.11 ± 3.98	6.81 ± 4.12	0.466
	36–38 GW	(kg)	12.32 ± 5.45	8.92 ± 6.1	0.001
Systolic blood pressure		(mmHg)	107.94 ± 10.9	111.44 ± 10.09	0.002
Diastolic blood pressure		(mmHg)	66.65 ± 8.72	67.22 ± 8.02	0.536
Fasting blood glucose		(mg/dL)	80.24 ± 6.02	82.41 ± 5.35	0.001
HbA1c		(%)	5.12 ± 0.22	5.27 ± 0.11	0.084
Family history	DM	*n* (%)	18 (3.8)	9 (7.1)	0.557
	MetS	*n* (%)	116 (24.3)	27 (21.3)	
Gestational history	None	*n* (%)	262 (54.8)	64 (50.4)	0.001
	GDM	*n* (%)	19 (4)	5 (3.9)	
	Miscarriage	*n* (%)	175 (36.6)	46 (36.2)	
	Hypertension	*n* (%)	7 (1.5)	1 (0.8)	
Primiparous		(%)	127 (31.7)	32 (29.9)	0.727
Smoker	Never	(%)	292 (88.8)	78 (94)	0.160
Ethnicity	Caucasian	(%)	309 (64.4)	79 (63.7)	0.855
	Latin American	(%)	163 (34)	42 (33.9)	
	Others	(%)	8 (1.7)	3 (2.4)	
MEDAS score	12 GW	(score)	5.00 ± 1.80	5.17 ± 1.73	0.349
	24 GW	(score)	5.60 ± 1.89	5.77 ± 1.78	0.418

NTG, Normal Glucose Tolerance; DM, Diabetes Mellitus; MetS, Metabolic Syndrome; GDM, Gestational Diabetes Mellitus; GW, gestational weeks; BMI, body mass index.

Dietary patterns during pregnancy were comparable between groups and did not influence the development of GDM. Similarly, no significant dietary differences were observed between groups at 6 years postpartum (Supplementary Table 1). Overall, adherence to the Mediterranean diet remained low throughout the study period. Regarding physical activity, no significant differences were found between groups 6 years postpartum (Supplementary Table 1). Although women with prior GDM reported slightly lower weekly light-intensity physical activity, total physical activity levels, expressed as MET-min/week, were comparable between groups.

**Figure 3:**
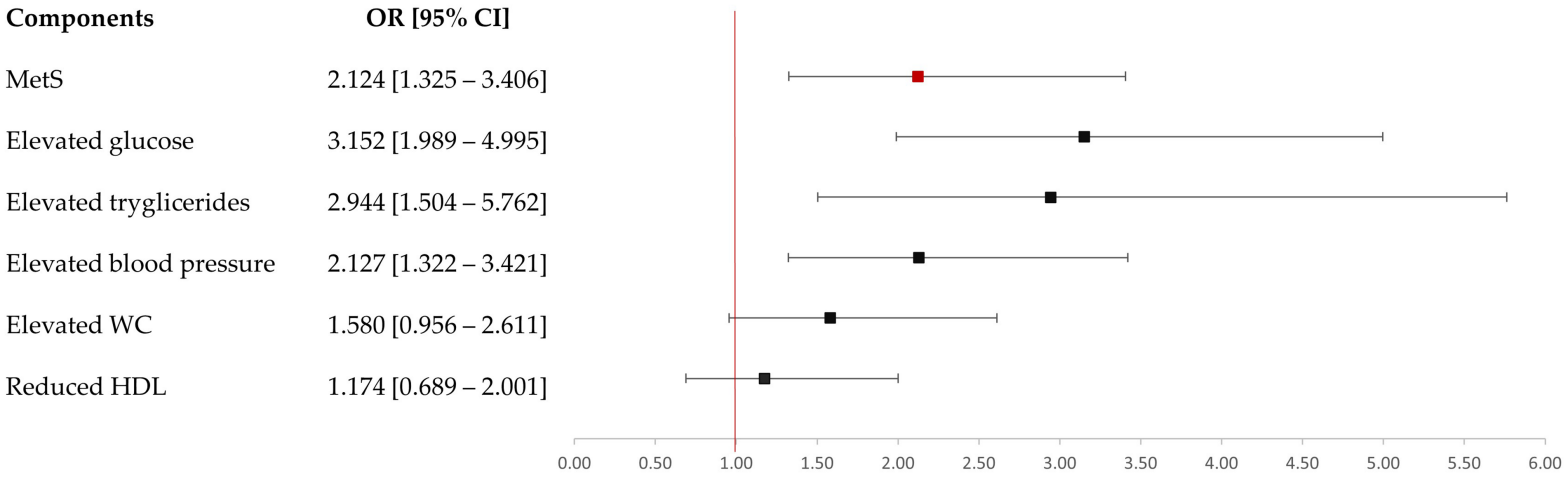
Risk of metabolic syndrome at six years postpartum by prior GDM.

Tables 2, 3 and Figure 3 summarize the assessment of MetS, anthropometric and body composition variables and MetS risk at 6 years postpartum according to previous GDM diagnosis.

**Table 2:** Metabolic syndrome at 6 years postpartum stratified by prior GDM diagnosis (Mean ± SD).

		NGT (*n* = 480)	GDM (*n* = 124)	*P*-value
MetS	*n* (%)	70 (14.6)	33 (26.6)	0.001
0 Criteria	*n* (%)	172 (35.8)	32 (25.8)	0.002
1 Criterion	*n* (%)	165 (34.4)	36 (29.0)	
2 Criteria	*n* (%)	73 (15.2)	23 (18.5)	
3 Criteria	*n* (%)	43 (9.0)	16 (12.9)	
4 Criteria	*n* (%)	21 (4.4)	9 (7.3)	
5 Criteria	*n* (%)	6 (1.3)	8 (6.5)	
MetS criteria				
Elevated WC	*n* (%)	69 (14.4)	26 (21)	0.072
Waist circumference	(cm)	78.46 ± 9.76	81.01 ± 11.24	0.012
Elevated triglycerides	*n* (%)	23 (4.8)	16 (12.9)	0.001
Triglycerides	(mg/dL)	77.47 ± 37.03	95.76 ± 56.49	0.001
Reduced HDL	*n* (%)	71 (14.8)	21 (16.9)	0.554
HDL cholesterol	(mg/dL)	62.24 ± 12.17	61.42 ± 11.7	0.501
Elevated blood pressure	*n* (%)	68 (14.8)	33 (27)	0.002
Systolic blood pressure	(mmHg)	107.25 ± 12.41	108.65 ± 12.00	0.261
SBP ≥ 130 mmHg	*n* (%)	28 (5.8)	5 (4)	0.431
Diastolic blood pressure	(mmHg)	77.48 ± 8.49	79.01 ± 9.27	0.081
DBP ≥ 85 mmHg	*n* (%)	84 (17.5)	32 (25.8)	0.036
Elevated FPG	n (%)	63 (13.1)	40 (32.3)	0.001
Glucose	(mg/dL)	91.45 ± 6.69	96.98 ± 8.94	0.001

NTG, Normal Glucose Tolerance; GDM, Gestational Diabetes Mellitus; WC, waist circumference.

**Table 3:** Anthropometric and body composition parameters at 6 years postpartum stratified by prior gestational diabetes mellitus diagnosis (Mean ± SD).

		NGT (*n* = 480)	GDM (*n* = 124)	*P*-value
Age	(years)	40.13 ± 5.19	41.98 ± 4.86	0.003
Weight	(kg)	64.82 ± 11.45	66.72 ± 12.31	0.105
WHtR		0.48 ± 0.07	0.50 ± 0.08	0.006
WHtR risk	*n* (%)	174 (36.3)	51 (41.1)	0.324
FM	(kg)	23.39 ± 8.25	24.66 ± 8.52	0.131
	(%)	35.12 ± 6.91	36.02 ± 6.67	0.193
FMI	(kg/m^2^)	8.9 ± 3.17	9.49 ± 3.38	0.070
Visceral fat	(L)	1.13 ± 0.67	1.25 ± 0.71	0.075
FFM	(kg)	41.39 ± 4.38	42.03 ± 4.79	0.158
FFM index	(kg/m^2^)	15.92 ± 1.45	16.24 ± 1.76	0.069
FM/FFM		0.56 ± 0.17	0.58 ± 0.17	0.234
SMM	(kg)	17.99 ± 2.04	18.16 ± 2.44	0.439
SMMI	(kg/m^2^)	6.83 ± 0.68	6.96 ± 0.82	0.057
SMM/weight	(%)	28.26 ± 3.77	27.66 ± 3.13	0.117
ASM	(kg)	14.93 ± 1.82	15.24 ± 2.04	0.099
ASMI	(kg/m^2^)	5.67 ± 0.63	5.85 ± 0.74	0.013
ASM/BMI		0.61 ± 0.07	0.6 ± 0.07	0.103
TBW	(L)	30.81 ± 3.39	31.22 ± 3.86	0.239
	(%)	47.92 ± 4.88	47.35 ± 4.6	0.245
ECW	(L)	13.56 ± 1.53	13.74 ± 1.67	0.323
ECW/TBW		0.45 ± 0.02	0.45 ± 0.03	0.223
Phase angle	(°)	5.04 ± 0.48	5.12 ± 0.51	0.087
PhA/BMI		0.21 ± 0.03	0.20 ± 0.03	0.202
Resistance	(ohm)	679.54 ± 72.75	662.87 ± 81.01	0.027
Reactance	(ohm)	59.67 ± 7.08	59.69 ± 7.48	0.973
HGS	(kg)	28.13 ± 4.76	27.34 ± 4.69	0.102
HGS/BMI		1.16 ± 0.26	1.09 ± 0.26	0.008

NTG, Normal Glucose Tolerance; GDM, Gestational Diabetes mellitus; FM, Fat Mass; FFM, Fat Free Mass; WHtR, waist-to-height ratio; SMM, Skeletal muscle mass; SMMI, Skeletal muscle mass index; SMI, Skeletal muscle index; ASM, Appendicular skeletal muscle mass; ASMI, Appendicular skeletal muscle mass index; TBW, Total Body Water; ECW, extracellular water; TBW, total body water; HGS, Handgrip strength.

At 6 years postpartum, 26.6% of women who had developed GDM during pregnancy were diagnosed with MetS (Table 2). Notably, these women had a 2-fold increased risk of developing MetS over these years compared to their normoglycemic counterparts (Figure 3). They also presented a higher frequency of ≥3 MetS diagnostic criteria (*p* = 0.001). However, 14.6% women exhibiting normal glucose tolerance (NGT) also developed MetS during the follow-up period. The *post-hoc* power to detect the difference between GDM and NGT was 85.7% (*p* < 0.05).

Among the biochemical components for the diagnosis of MetS, elevated triglycerides and FPG levels were significantly influenced by the prior GDM diagnosis, whereas low HDL cholesterol concentration was not. These two elevated diagnostic criteria showed an increased risk over the 6 years postpartum in women with previous GDM diagnosis. Notably, several parameters related to glucose metabolism and lipid profile were affected by a prior diagnosis of GDM. However, mean values for these parameters remained within normal reference ranges for all the study population. Furthermore, safety parameters were comparable across all participants, with none exceeding established clinical normal ranges (Supplementary Table 2). Additionally, the hypertension risk was also increased in these women, with DBP appearing to be more conditioned by this diabetogenic condition.

Conversely, although the prior GDM diagnosis during pregnancy entailed a greater WC compared to NGT women, there were no significant differences when a risk circumference was evaluated (> 89.5 cm). Indeed, the presence of GDM did not significantly affect the risk of meeting this anthropometric criterion for MetS diagnosis over time. Similarly, body weight at 6 years postpartum was comparable between women who were previously diagnosed with GDM versus those who were not (Table 3). Moreover, a history of GDM was not significantly associated with the presence of an elevated body mass index (BMI ≥ 25 kg/m^2^; OR = 1.254 [0.689–2.001]) or with an elevated WHtR risk (OR = 1.225 [0.818–1.833]) over time. On the other hand, body composition at 6 years postpartum was poorly affected by previous GDM diagnosis during pregnancy (Table 4). FM, visceral fat or FFM were similar between women with prior GDM diagnosis or without. SMMI and ASMI were higher in women who had developed GDM. Among the raw data parameters, only resistance was significantly affected by prior GDM diagnosis, presenting lower values. Nevertheless, these differences were no longer evident when body composition was assessed after stratification by previous GDM diagnosis and MetS diagnosis (Supplementary Table 3). However, body composition was significantly different when it was only assessed depending on the presence or absence of MetS at 6 years postpartum (Table 4).

**Table 4:** Anthropometric and body composition parameters stratified by Metabolic Syndrome at 6 years Postpartum (Mean ± SD).

		No MetS (*n* = 501)	MetS (*n* = 103)	*p*-value
Age	(years)	39.90 ± 5.29	42.28 ± 4.76	0.001
Weight	(kg)	62.38 ± 9.57	78.91 ± 11.14	0.001
WHtR		0.47 ± 0.05	0.58 ± 0.06	0.001
WHtR risk	*n* (%)	134 (26.5)	92 (90.2)	0.001
FM	(kg)	21.53 ± 6.66	33.97 ± 7.85	0.001
	(%)	33.84 ± 6.19	42.51 ± 5.31	0.001
FMI	(kg/m^2^)	8.18 ± 2.54	13.16 ± 3.04	0.001
Visceral fat	(L)	1.00 ± 0.55	1.97 ± 0.69	0.001
FFM	(kg)	40.92 ± 4.26	44.52 ± 4.32	0.001
FFMI	(kg/m^2^)	15.68 ± 1.31	17.52 ± 1.58	0.001
FM/FFM		0.52 ± 0.14	0.76 ± 0.15	0.001
SMM	(kg)	17.80 ± 2.04	19.13 ± 2.22	0.001
SMMI	(kg/m^2^)	6.74 ± 0.63	7.39 ± 0.82	0.001
SMM/weight	(%)	28.90 ± 3.51	24.38 ± 2.35	0.001
ASM	(kg)	14.62 ± 1.63	16.82 ± 1.9	0.001
ASMI	(kg/m^2^)	5.54 ± 0.52	6.5 ± 0.68	0.001
ASM/BMI		0.62 ± 0.07	0.54 ± 0.06	0.001
TBW	(L)	30.41 ± 3.34	33.31 ± 3.22	0.001
	(%)	48.85 ± 4.35	42.72 ± 3.66	0.001
ECW	(L)	13.45 ± 1.5	14.76 ± 1.28	0.001
ECW/TBW		0.45 ± 0.02	0.45 ± 0.02	0.895
Phase angle	(°)	5.00 ± 0.47	5.29 ± 0.53	0.001
PhA/BMI		0.21 ± 0.03	0.17 ± 0.03	0.001
Resistance	(ohm)	687.68 ± 70.16	620.23 ± 71.25	0.001
Reactance	(ohm)	60.15 ± 7.1	57.37 ± 6.99	0.001
HGS	(kg)	27.99 ± 4.79	27.6 ± 4.8	0.457
HGS/BMI		1.19 ± 0.25	0.90 ± 0.20	0.001

MetS, Metabolic Syndrome; FM, Fat Mass; FFM, Fat Free Mass; WHtR, waist-to-height ratio; SMM, Skeletal muscle mass; SMMI, Skeletal muscle mass index; SMI, Skeletal muscle index; ASM, Appendicular skeletal muscle mass; ASMI, Appendicular skeletal muscle mass index; TBW, Total Body Water; ECW, extracellular water; TBW, total body water; HGS, Handgrip strength.

In this context, women with MetS exhibited significantly greater adiposity in both absolute (FM, visceral fat) and relative terms (FMI, FM/FFM). Although these women also had higher absolute muscle mass (SMM, ASM) their muscle mass relative to body size (SMM/weight, ASM/BMI ratio) was significantly lower compared to those with no MetS at 6 years postpartum. Notably, TBW and ECW were also higher in women with MetS diagnosis, however, the proportion of total water relative to weight (TBW, %) was lower, evidencing a higher relative FM. Notably, the ECW/TBW ratio did not differ significantly between groups (*p* = 0.895), further supporting the notion of increased adiposity rather than altered fluid distribution.

This excess adiposity was also associated with significantly lower values of resistance and reactance in women with MetS. Whereas PhA, a marker of cellular integrity and function, was higher in women with MetS, it was significantly lower when adjusted for BMI (PhA/BMI), suggesting inadequate cellular health relative to body mass. A similar pattern occurs when muscular strength was observed, although absolute HGS was comparable between groups, functional strength relative to body weight (HGS/BMI) was substantially lower in women with MetS at 6 years postpartum.

When diagnostic performance was evaluated, variables that demonstrated the highest diagnostic capacity for MetS-with AUC values ≥ 0.85 (very good discrimination)-were FM (kg, %), visceral fat, FM/FFM ratio, and ASM. In contrast, SMM/weight’ and TBW’ (%) exhibited strong discriminatory power for discarding MetS (Table 5).

**Table 5:** Diagnostic performance of body composition parameters to discriminate metabolic syndrome.

		AUC [95% CI]	Youden index	Cut-off	Sensitivity (%)	Specificity (%)	PPV (%)	NPV (%)
FM	(kg)	0.89 [0.85–0.92]	0.602	26.43	81	80	45	95
	(%)	0.86 [0.82–0.90]	0.575	37.15	89	69	38	97
FMI	(kg/m^2^)	0.90 [0.87–0.93]	0.650	9.53	91	74	42	98
Visceral fat	(L)	0.86 [0.82–0.90]	0.582	1.45	80	78	41	95
FFM	(kg)	0.73 [0.68–0.78]	0.364	40.91	82	54	27	94
FFM intex	(kg/m^2^)	0.83 [0.79–0.87]	0.519	16.89	70	83	46	93
FM/FFM ratio		0.87 [0.83–0.90]	0.581	0.593	88	70	37	97
SMM	(kg)	0.68 [0.62–0.73]	0.277	18.01	71	57	26	90
SMMI	(kg/m^2^)	0.73 [0.67–0.78]	0.347	7.25	52	82	36	89
SMM/weight	(%)	0.87 [0.83–0.90]	0.593	27.01	71	89	39	96
ASM	(kg)	0.82 [0.77–0.86]	0.492	15.78	70	80	42	93
ASMI	(kg/m^2^)	0.88 [0.84–0.91]	0.634	5.97	82	82	49	96
ASM/BMI		0.78 [0.74–0.83]	0.445	0.603	87	58	30	95
Total body water	(L)	0.74 [0.69–0.79]	0.381	30.75	81	57	28	94
Total body water	(%)	0.86 [0.82–0.90]	0.566	44.95	75	82	47	94
Extracellular water	(L)	0.76 [0.70–0.82]	0.480	13.85	80	68	30	95
Phase angle	(°)	0.67 [0.61–0.73]	0.292	5.05	74	56	9	74
PhA/BMI		0.85 [0.81–0.89]	0.585	0.184	83	75	6	52
Resistance	(ohm)	0.75 [0.70–0.80]	0.388	643.65	66	74	35	91
Reactance	(ohm)	0.62 [0.56–0.68]	0.219	58.25	61	61	25	88

FM, fat mass; FFM, Fat Free Mass; SMM, Skeletal muscle mass; SMMI, Skeletal muscle mass index; ASM, Appendicular skeletal muscle mass; ASMI, Appendicular skeletal muscle mass index; Model vs. no MetS (values above the cut-off point indicate reduced risk of MetS).

Although the models demonstrated good sensitivity and specificity, the relatively low prevalence of MetS in the cohort (17%) limited the positive predictive value (PPV), which ranged from 25 to 49%. In contrast, negative predictive values (NPV) were consistently high (≥90%) in almost all body composition parameters. Therefore, these diagnostic tests were particularly useful for excluding the presence of MetS in women at 6 years postpartum.

Moreover, these findings underscore the strong relationship between body composition and MetS. The variables that showed the greatest differences between women with and without MetS were also the most effective as screening tools, indicating a robust internal consistency between diagnostic capacity and group differences in body composition.

## 4 Discussion

The results of this study confirm GDM as an important risk factor for MetS; however, it also highlights that it does not equally impact over the distinct diagnostic criteria. Specifically, elevated WC did not represent a MetS risk factor in these women. In contrast, body composition, specifically, high fat mass and low muscle content and function, showed a strong association with the presence of MetS. Therefore, the findings of this study show that assessment of body composition, particularly adiposity, emerges as an important complementary tool to anthropometric parameters in the detection of MetS in postpartum women.

GDM represents one of the most prevalent metabolic complications during pregnancy, and it is associated with multiple maternal and perinatal risk factors (43, 44). Consistent with the literature (45), in the present study, advanced maternal age was higher in women who were later diagnosed with GDM. Likewise, pregestational body weight and BMI were significantly higher, with a higher proportion of GDM women with BMI ≥ 25 kg/m^2^. This association reinforces the evidence that a high pregestational body mass is determinant in the pathogenesis of GDM (44, 46). In this regard, some authors have shown that a high prepregnancy BMI represents an even stronger risk factor than advanced maternal age for the development of GDM (47). Weight gain towards the end of the pregnancy was lower in women with GDM, which was expected due to the tight monitoring of weight in women with prepregnancy overweight, highly prevalent in these women. Therefore, from the beginning of pregnancy, excess weight determines metabolic changes.

After 6 years postpartum, data indicate a significantly higher prevalence of MetS among women with a prior diagnosis of GDM. These women exhibited a two-fold increased risk of developing MetS, although this risk estimate is somewhat lower than that reported in a recent meta-analysis, which found a three-fold increase (48). This finding reinforces the evidence that GDM is a strong predictor of future metabolic dysfunction (48–51). Nonetheless, the findings of the present study indicate that the association is not uniform across the different diagnostic criteria for MetS. In women with prior diagnosis of GDM, an increased WC did not emerge as an independent risk factor for MetS and exhibited values comparable to those of normoglycemic counterparts. This is particularly relevant because, although there is no mandatory criterion for the diagnosis of MetS, WC measurement is still recommended as a preliminary screening tool (22). Other anthropometric parameters linked to cardiometabolic risk, as elevated BMI and WHtR, or even body weight, were likewise unaffected by prior diagnosis of GDM. This suggests that the evaluation of body composition may provide a more accurate assessment of MetS risk relative to conventional anthropometric parameters, despite the discriminatory capability of central adiposity measures in detecting cardiometabolic risk (52–54). In this context, an expanding amount of evidence indicates that a precise characterization of the body distribution, rather than isolated anthropometric parameters, is essential to better understand and assess of health status (55–58).

At 6 years postpartum, body composition parameters did not differ significantly between women with a history of GDM and those without. This finding suggests that pregnancy itself-particularly when accompanied by greater gestational weight gain and continued weight gain over time, as observed among women who did not develop GDM-may induce hormonal, inflammatory, and/or metabolic alterations that persist long-term (59–61). This could explain the convergence in body composition parameters in these women over time, irrespective of GDM diagnosis during pregnancy. In contrast, when stratified by the presence of MetS, notable differences were observed. Consistent with findings from previous studies (62, 63), women who met the diagnostic criteria for MetS at 6 years postpartum exhibited significantly less favorable body composition parameters compared to those without MetS. This condition was marked by increased adiposity, both in absolute terms (FM and visceral fat)-the latter approximately twice in individuals with MetS-and in adjusted measures (FMI and FM/FFM). These findings were supported by a lower hydration status relative to body weight (TBW, %), suggesting a higher adipose tissue proportion. Absolute estimators of muscle mass such as SMM and ASM, also including FFM, were higher in women with MetS, likely as consequence of greater overall body weight. These findings are in line with previous reports that have documented similar associations between increased muscle mass parameters and MetS in female populations (64). However, several studies have indicated that muscle mass parameters adjusted for body size provide more accurate information of cardiometabolic risk (65–68). Consistently, women with MetS exhibited lower SMM/weight and ASM/BMI, as well as reduced functional performance (HGS/BMI) and compromised cellular health (PhA/BMI) when these were normalized for body size. Resistance and reactance were significantly lower in MetS, possibly due to higher FM and a lower cell integrity (69). These body composition alterations suggest a heightened metabolic and functional risk that may not be apparent when relying exclusively on conventional anthropometric parameters (70).

These findings provided a rationale for the development of predictive models to assess the potential usefulness of body composition parameters in the identification of MetS and, for the first time, the establishment of specific cut-off values for their identification. In this context, adipose compartments mainly FM, visceral fat, FMI and FM/FFM, had an excellent diagnostic capacity for MetS (AUC ≥ 85%). FMI and FM (kg) exhibit also an adequate balance between sensitivity and specificity, supporting their potential utility in the development of predictive models or screening algorithms of MetS. The identified cut-off values for the different body composition parameters were also quite similar to those established for other clinical conditions, such as obesity (FM > 39%) (71) or sarcopenia-reduced muscle mass (SMM/Weight < 27.6%) (33), low muscle mass (ASM < 15 kg; ASMI < 5.5 kg/m^2^) (72)-demonstrating consistency in the data found.

Nevertheless, from a clinical perspective, the low PPV observed reflects a substantial rate of false-positive results following test administration. These findings are consistent with the low prevalence of MetS in the population (17%), a factor that adversely affects PPV, even when test sensitivity and specificity are high (73, 74). In return, the post-test results revealed a high NPV, indicating that, in clinical practice, these diagnostic tests are particularly effective for dismissing the presence of MetS in postpartum women when test results are negative.

The present findings represent a significant step forward in the clinical application of body composition analysis, as they have allowed, for the first time, the definition of specific cut-off values for identifying individuals at risk of MetS. These cut-offs not only enhance the understanding of the pathophysiological link between altered body composition and MetS, but also provide a robust basis for the development of predictive models. Such models may improve the diagnostic accuracy and clinical utility of body composition parameters, highlighting their potential as accessible, non-invasive screening tools for early detection and risk stratification in routine clinical practice. This highlights their utility not only for identifying individuals at risk, but also for reassuring those unlikely to be affected, thus optimizing resource allocation and follow-up strategies in clinical settings.

This study has some limitations that merit consideration. Its cross-sectional design limits the ability to establish causal relationships between body composition indicators and the presence of MetS. Although BIA was used for its accessibility and non-invasive nature, it is less precise than reference methods such as DXA or MRI, particularly in individuals with an altered hydration status or high adiposity. Furthermore, the proposed cut-off points have not been validated in external cohorts, which may limit their generalizability and may restrict their applicability to other populations. Lastly, the influence of potential confounding variables, such as physical activity, dietary patterns, and comorbidities, was not fully addressed, potentially affecting the observed associations.

## 5 Conclusion

This study confirms GDM as a significant long-term risk factor for MetS in postpartum women. However, the association is not uniform across diagnostic criteria, with WC showing limited discriminative value. In contrast, body composition, particularly increased adiposity and reduced relative muscle mass and function, showed a stronger association with MetS. These results underscore the added clinical value of body composition assessment over conventional anthropometric measures. Additionally, the proposed cut-off values for key parameters demonstrated strong diagnostic performance, especially for fat-related indices. Although positive predictive value was limited by the low MetS prevalence, the high NPV supports its utility in excluding MetS. Overall, body composition analysis emerges as a valuable complementary tool for improving cardiometabolic risk assessment in postpartum care.

## Data Availability

The datasets presented in this study can be found in online repositories. The names of the repository/repositories and accession number(s) can be found at: https://doi.org/10.5281/zenodo.15586793.

## Footnotes

^1^ https://www.idissc.org/estudios-clinicos-y-preclinicos/contacto-ceim/

## References

[1] ElSayed NA McCoy RG Aleppo G Balapattabi K Beverly EA Briggs Early K. Diagnosis and classification of diabetes: standards of care in diabetes—2025. Diabetes Care. (2025) 48:S27–49. doi: 10.2337/DC25-S002, PMID: 39651986 PMC11635041

[2] World Health Organization. Diagnostic criteria and classification of hyperglycaemia first detected in pregnancy: a world health organization guideline. Diabetes Res Clin Pract. (2014) 103:341–63. doi: 10.1016/j.diabres.2013.10.012 24847517

[3] Wang H Li N Chivese T Werfalli M Sun H Yuen L. IDF diabetes atlas: estimation of global and regional gestational diabetes mellitus prevalence for 2021 by International Association of Diabetes in pregnancy study group’s criteria. Diabetes Res Clin Pract. (2022) 183:109050. doi: 10.1016/j.diabres.2021.109050, PMID: 34883186

[4] De La Torre NG Assaf-Balut C Varas IJ Del Valle L Durán A Fuentes M. Effectiveness of following Mediterranean diet recommendations in the real world in the incidence of gestational diabetes mellitus (GDM) and adverse maternal-foetal outcomes: a prospective, universal, interventional study with a single group. The St Carlos study. Nutrients. (2019) 11:1210. doi: 10.3390/NU11061210 31141972 PMC6627921

[5] Plows JF Stanley JL Baker PN Reynolds CM Vickers MH. The pathophysiology of gestational diabetes mellitus. Int J Mol Sci. (2018) 19:3342. doi: 10.3390/IJMS19113342, PMID: 30373146 PMC6274679

[6] Nguyen B Tselovalnikova T Drees BM. Gestational diabetes mellitus and metabolic syndrome: a review of the associations and recommendations. Endocr Pract. (2024) 30:78–82. doi: 10.1016/J.EPRAC.2023.10.133, PMID: 37918624

[7] Sheiner E. Gestational diabetes mellitus: long-term consequences for the mother and child grand challenge: how to move on towards secondary prevention? Front Clin Diabetes Healthc. (2020) 1:546256. doi: 10.3389/FCDHC.2020.546256, PMID: 36993989 PMC10041873

[8] Pathirana MM Lassi ZS Ali A Arstall MA Roberts CT Andraweera PH. Association between metabolic syndrome and gestational diabetes mellitus in women and their children: a systematic review and meta-analysis. Endocrine. (2021) 71:310–20. doi: 10.1007/S12020-020-02492-1, PMID: 32930949

[9] Burlina S Dalfrà MG Chilelli NC Lapolla A. Gestational diabetes mellitus and future cardiovascular risk: an update. Int J Endocrinol. (2016) 2016:1–6. doi: 10.1155/2016/2070926, PMID: 27956897 PMC5124460

[10] Lim S Sung HC Young JP Kyong SP Hong KL Jang HC. Visceral fatness and insulin sensitivity in women with a previous history of gestational diabetes mellitus. Diabetes Care. (2007) 30:348–53. doi: 10.2337/DC06-1405 17259506

[11] Svensson H Wetterling L Andersson-Hall U Jennische E Edén S Holmäng A. Adipose tissue and body composition in women six years after gestational diabetes: factors associated with development of type 2 diabetes. Adipocyte. (2018) 7:229–37. doi: 10.1080/21623945.2018.1521230, PMID: 30246599 PMC6768253

[12] Meyer D Gjika E Raab R Michel SKF Hauner H. How does gestational weight gain influence short- and long-term postpartum weight retention? An updated systematic review and meta-analysis. Obes Rev. (2024) 25:e13679. doi: 10.1111/OBR.13679, PMID: 38221780

[13] Berezowsky A Berger H. Gestational weight gain and long-term postpartum weight retention. Clin Exp Obstet Gynecol. (2021) 48:466–71. doi: 10.31083/J.CEOG.2021.03.2413/0390-6663-48-3-466.PDF

[14] Kramer CK Ye C Hanley AJ Connelly PW Sermer M Zinman B. Postpartum weight retention and the early evolution of cardiovascular risk over the first 5 years after pregnancy. Cardiovasc Diabetol. (2024) 23:1–10. doi: 10.1186/S12933-024-02184-4/FIGURES/1 38500162 PMC10949683

[15] Després JP. Body fat distribution and risk of cardiovascular disease: an update. Circulation. (2012) 126:1301–13. doi: 10.1161/CIRCULATIONAHA.111.067264 22949540

[16] Duran A Śaenz S Torrej́on MJ Bordí UE Del Valle L Galindo M. Introduction of IADPSG criteria for the screening and diagnosis of gestational diabetes mellitus results in improved pregnancy outcomes at a lower cost in a large cohort of pregnant women: the St. Carlos gestational diabetes study. Diabetes Care. (2014) 37:2442–50. doi: 10.2337/DC14-0179 24947793

[17] Del VL Melero V Bodas A O’Connor RM Ramos-Levi A Barabash A. A greater adherence to the Mediterranean diet supplemented with extra virgin olive oil and nuts during pregnancy is associated with improved offspring health at six years of age. Forum Nutr. (2025) 17:1719. doi: 10.3390/NU17101719 PMC12113803 40431459

[18] WMA Declaration of Helsinki – Ethical principles for medical research involving human participants – WMA – the world medical association. Available online at: https://www.wma.net/policies-post/wma-declaration-of-helsinki/ (Accessed May 26, 2025). 10.1001/jama.2024.21972 39425955

[19] Medicines Agency E. ICH E6 (R3) Guideline on good clinical practice (GCP)_Step 5. Available online at: www.ema.europa.eu/contact (Accessed May 26, 2025).

[20] IADPSGC. National Institutes of Health consensus development conference statement: diagnosing gestational diabetes mellitus, March 4-6, 2013. Obstet Gynecol. (2013) 122:358–69. doi: 10.1097/AOG.0B013E31829C3E64 23969806

[21] Metzger BE. International association of diabetes and pregnancy study groups recommendations on the diagnosis and classification of hyperglycemia in pregnancy. Diabetes Care. (2010) 33:676–82. doi: 10.2337/DC09-1848 20190296 PMC2827530

[22] Alberti KGMM Eckel RH Grundy SM Zimmet PZ Cleeman JI Donato KA. Harmonizing the metabolic syndrome: a joint interim statement of the international diabetes federation task force on epidemiology and prevention; national heart, lung, and blood institute; American heart association; world heart federation; international atherosclerosis society; and international association for the study of obesity. Circulation. (2009) 120:1640–5. doi: 10.1161/CIRCULATIONAHA.109.192644, PMID: 19805654

[23] Marcuello C Calle-Pascual AL Fuentes M Runkle I Rubio MA Montañez C. Prevalence of the metabolic syndrome in Spain using regional cutoff points for waist circumference: the di@bet.es study. Acta Diabetol. (2013) 50:615–23. doi: 10.1007/S00592-013-0468-8, PMID: 23512475

[24] FAO/UNICEF/WHO. Methodology of nutritional surveillance. Report of a joint FAO/UNICEF/WHO expert committee - PubMed. Available online at: https://pubmed.ncbi.nlm.nih.gov/822593/ (Accessed May 26, 2025).

[25] Norton K Whittingham N Carter L Kerr D Gore CM-JM. Antropometrica. In: Norton KOT, editor. Measurement techniques in anthropometry. Sydney: UNSW (1966). 27–75.

[26] Gibson S Ashwell M. A simple cut-off for waist-to-height ratio (0·5) can act as an indicator for cardiometabolic risk: recent data from adults in the health survey for England. Br J Nutr. (2020) 123:681–90. doi: 10.1017/S0007114519003301, PMID: 31840619

[27] Browning LM Hsieh SD Ashwell M. A systematic review of waist-to-height ratio as a screening tool for the prediction of cardiovascular disease and diabetes: 05 could be a suitable global boundary value. Nutr Res Rev. (2010) 23:247–69. doi: 10.1017/S0954422410000144, PMID: 20819243

[28] Kyle UG Bosaeus I De Lorenzo AD Deurenberg P Elia M Gómez JM. Bioelectrical impedance analysis - part I: review of principles and methods. Clin Nutr. (2004) 23:1226–43. doi: 10.1016/j.clnu.2004.06.004, PMID: 15380917

[29] Kyle UG Bosaeus I De Lorenzo AD Deurenberg P Elia M Gómez JM. Bioelectrical impedance analysis - part II: utilization in clinical practice. Clin Nutr. (2004) 23:1430–53. doi: 10.1016/j.clnu.2004.09.012, PMID: 15556267

[30] Bosy-Westphal A Schautz B Later W Kehayias JJ Gallagher D Müller MJ. What makes a BIA equation unique? Validity of eight-electrode multifrequency BIA to estimate body composition in a healthy adult population. Eur J Clin Nutr. (2013) 67:S14–21. doi: 10.1038/EJCN.2012.160, PMID: 23299866

[31] VanItallie TB Yang MU Heymsfield SB Funk RC Boileau RA. Height-normalized indices of the body’s fat-free mass and fat mass: potentially useful indicators of nutritional status. Am J Clin Nutr. (1990) 52:953–9. doi: 10.1093/ajcn/52.6.953, PMID: 2239792

[32] Xiao J Purcell SA Prado CM Gonzalez MC. Fat mass to fat-free mass ratio reference values from NHANES III using bioelectrical impedance analysis. Clin Nutr. (2018) 37:2284–7. doi: 10.1016/j.clnu.2017.09.021, PMID: 29056283

[33] Janssen I Heymsfield SB Ross R. Low relative skeletal muscle mass (sarcopenia) in older persons is associated with functional impairment and physical disability. J Am Geriatr Soc. (2002) 50:889–96. doi: 10.1046/J.1532-5415.2002.50216.X, PMID: 12028177

[34] Sergi G De Rui M Veronese N Bolzetta F Berton L Carraro S. Assessing appendicular skeletal muscle mass with bioelectrical impedance analysis in free-living Caucasian older adults. Clin Nutr. (2015) 34:667–73. doi: 10.1016/j.clnu.2014.07.010, PMID: 25103151

[35] Gould H Brennan SL Kotowicz MA Nicholson GC Pasco JA. Total and appendicular lean mass reference ranges for Australian men and women: the Geelong osteoporosis study. Calcif Tissue Int. (2014) 94:363–72. doi: 10.1007/S00223-013-9830-7, PMID: 24390582

[36] Bahat G Kilic C Ilhan B Karan MA Cruz-Jentoft A. Association of different bioimpedanciometry estimations of muscle mass with functional measures. Geriatr Gerontol Int. (2019) 19:593–7. doi: 10.1111/GGI.13668, PMID: 31006968

[37] Mathiowetz V Weber K Volland G Kashman N. Reliability and validity of grip and pinch strength evaluations. J Hand Surg Am. (1984) 9:222–6. doi: 10.1016/S0363-5023(84)80146-X, PMID: 6715829

[38] Mathiowetz V Kashman N Volland G Weber K Dowe M Rogers S. Grip and pinch strength: normative data for adults. Arch Phys Med Rehabil. (1985) 66:69–74. 3970660

[39] McGrath R Hackney KJ Ratamess NA Vincent BM Clark BC Kraemer WJ. Absolute and body mass index normalized handgrip strength percentiles by gender, ethnicity, and hand dominance in Americans. Adv Geriatr Med Res. (2019) 2:e200005. doi: 10.20900/AGMR20200005, PMID: 31930203 PMC6954001

[40] Schröder H Fitó M Estruch R Martínez-González MA Corella D Salas-Salvadó J. A short screener is valid for assessing mediterranean diet adherence among older Spanish men and women. J Nutr. (2011) 141:1140–5. doi: 10.3945/jn.110.135566, PMID: 21508208

[41] Martínez-González MA García-Arellano A Toledo E Salas-Salvadó J Buil-Cosiales P Corella D. A 14-item mediterranean diet assessment tool and obesity indexes among high-risk subjects: the PREDIMED trial. PLoS One. (2012) 7:e43134. doi: 10.1371/JOURNAL.PONE.0043134, PMID: 22905215 PMC3419206

[42] Booth M. Assessment of physical activity: an international perspective. Res Q Exerc Sport. (2000) 71:114–20. doi: 10.1080/02701367.2000.11082794 25680021

[43] ADA ADAPPC. 15. Management of diabetes in pregnancy: standards of medical care in diabetes—2022. Diabetes Care. (2022) 45:S232–43. doi: 10.2337/DC22-S015 34964864

[44] Zhang Y Xiao CM Zhang Y Chen Q Zhang XQ Li XF. Factors associated with gestational diabetes mellitus: a meta-analysis. J Diabetes Res. (2021) 2021:1–18. doi: 10.1155/2021/6692695, PMID: 34046504 PMC8128547

[45] Shirazian N Emdadi R Mahboubi M Motevallian A Fazel-Sarjuei Z Sedighpour N. Screening for gestational diabetes: usefulness of clinical risk factors. Arch Gynecol Obstet. (2009) 280:933–7. doi: 10.1007/S00404-009-1027-Y/METRICS 19301026

[46] Dong B Yu H Wei Q Zhi M Wu C Zhu X. The effect of pre-pregnancy body mass index and excessive gestational weight gain on the risk of gestational diabetes in advanced maternal age. Oncotarget. (2017) 8:58364–71. doi: 10.18632/ONCOTARGET.17651, PMID: 28938562 PMC5601658

[47] Mirabelli M Tocci V Donnici A Giuliano S Sarnelli P Salatino A. Maternal preconception body mass index overtakes age as a risk factor for gestational diabetes mellitus. J Clin Med. (2023) 12:2830. doi: 10.3390/JCM12082830, PMID: 37109166 PMC10145909

[48] Tranidou A Dagklis T Tsakiridis I Siargkas A Apostolopoulou A Mamopoulos A. Risk of developing metabolic syndrome after gestational diabetes mellitus - a systematic review and meta-analysis. J Endocrinol Investig. (2021) 44:1139–49. doi: 10.1007/S40618-020-01464-6, PMID: 33226626

[49] Akinci B Celtik A Yener S Yesil S. Prediction of developing metabolic syndrome after gestational diabetes mellitus. Fertil Steril. (2010) 93:1248–54. doi: 10.1016/j.fertnstert.2008.12.007, PMID: 19147138

[50] Aldridge E Pathirana M Wittwer M Sierp S Leemaqz SY Roberts CT. Prevalence of metabolic syndrome in women after maternal complications of pregnancy: an observational cohort analysis. Front Cardiovasc Med. (2022) 9:853851. doi: 10.3389/FCVM.2022.853851, PMID: 35360031 PMC8963931

[51] Zhao X Zhao D Sun J Yuan N Zhang X. Correlation between gestational diabetes mellitus and postpartum cardiovascular metabolic indicators and inflammatory factors: a cohort study of Chinese population. Front Endocrinol (Lausanne). (2024) 15:1401679. doi: 10.3389/FENDO.2024.1401679, PMID: 39655348 PMC11625572

[52] Lee CMY Huxley RR Wildman RP Woodward M. Indices of abdominal obesity are better discriminators of cardiovascular risk factors than BMI: a meta-analysis. J Clin Epidemiol. (2008) 61:646–53. doi: 10.1016/j.jclinepi.2007.08.012, PMID: 18359190

[53] Ashwell M Gunn P Gibson S. Waist-to-height ratio is a better screening tool than waist circumference and BMI for adult cardiometabolic risk factors: systematic review and meta-analysis. Obes Rev. (2012) 13:275–86. doi: 10.1111/J.1467-789X.2011.00952.X, PMID: 22106927

[54] Rico-Martín S Calderón-García JF Sánchez-Rey P Franco-Antonio C Martínez Alvarez M Sánchez Muñoz-Torrero JF. Effectiveness of body roundness index in predicting metabolic syndrome: a systematic review and meta-analysis. Obes Rev. (2020) 21:e13023. doi: 10.1111/OBR.13023, PMID: 32267621

[55] Holmes CJ Racette SB. The utility of body composition assessment in nutrition and clinical practice: an overview of current methodology. Nutrients. (2021) 13:2493. doi: 10.3390/NU13082493, PMID: 34444653 PMC8399582

[56] de Morais N d S Azevedo FM de Freitas Rocha AR Morais D d C Ribeiro SAV Gonçalves VSS. Body fat is superior to body mass index in predicting cardiometabolic risk factors in adolescents. Int J Environ Res Public Health. (2023) 20:2074. doi: 10.3390/IJERPH20032074/S1 36767439 PMC9915438

[57] Lee BJ Yim MH. Comparison of anthropometric and body composition indices in the identification of metabolic risk factors. Sci Rep. (2021) 11:9931. doi: 10.1038/S41598-021-89422-X, PMID: 33976292 PMC8113511

[58] Zeng Q Dong SY Sun XN Xie J Cui Y. Percent body fat is a better predictor of cardiovascular risk factors than body mass index. Braz J Med Biol Res. (2012) 45:591. doi: 10.1590/S0100-879X2012007500059, PMID: 22510779 PMC3854278

[59] Gunderson EP. Childbearing and obesity in women: weight before, during, and after pregnancy. Obstet Gynecol Clin N Am. (2009) 36:317–32. doi: 10.1016/J.OGC.2009.04.001, PMID: 19501316 PMC2930888

[60] Ishaku SM Karima T Oboirien KA Innocent AP Lawal O Jamilu T. Metabolic syndrome following hypertensive disorders in pregnancy in a low-resource setting: a cohort study. Pregnancy Hypertens. (2021) 25:129–35. doi: 10.1016/j.preghy.2021.05.018, PMID: 34119878

[61] Garcia De Leon R Hodges TE Brown HK Bodnar TS LAM G. Inflammatory signalling during the perinatal period: implications for short- and long-term disease risk. Psychoneuroendocrinology. (2025) 172:107245. doi: 10.1016/J.PSYNEUEN.2024.107245, PMID: 39561569

[62] Baudrand R Domínguez JM Tabilo C Figueroa D Jimenez M Eugenin C. The estimation of visceral adipose tissue with a body composition monitor predicts the metabolic syndrome. J Hum Nutr Diet. (2013) 26:154–8. doi: 10.1111/JHN.12089, PMID: 23634931

[63] Cho DH Kim MN Joo HJ Shim WJ Lim DS Park SM. Visceral obesity, but not central obesity, is associated with cardiac remodeling in subjects with suspected metabolic syndrome. Nutr Metab Cardiovasc Dis. (2019) 29:360–6. doi: 10.1016/j.numecd.2019.01.007, PMID: 30782509

[64] Pramyothin P Limpattanachart V Dawilai S Sarasak R Sukaruttanawong C Chaiyasoot K. Fat-free mass, metabolically healthy obesity, and type 2 diabetes in severely obese Asian adults. Endocr Pract. (2017) 23:915–22. doi: 10.4158/EP171792.OR, PMID: 28614006

[65] Kim TN Park MS Lee EJ Chung HS Yoo HJ Kang HJ. Comparisons of three different methods for defining sarcopenia: an aspect of cardiometabolic risk. Sci Rep. (2017) 7:6491. doi: 10.1038/S41598-017-06831-7, PMID: 28747657 PMC5529503

[66] Kim G Lee SE Jun JE Lee YB Ahn J Bae JC. Increase in relative skeletal muscle mass over time and its inverse association with metabolic syndrome development: a 7-year retrospective cohort study. Cardiovasc Diabetol. (2018) 17:23. doi: 10.1186/S12933-018-0659-2, PMID: 29402279 PMC5798183

[67] Song YM Lee K. Comparison of the associations between appendicular lean mass adjustment methods and cardiometabolic factors. Nutr Metab Cardiovasc Dis. (2020) 30:2271–8. doi: 10.1016/j.numecd.2020.07.036, PMID: 32980247

[68] Guan Z Minnetti M Heymsfield SB Poggiogalle E Prado CM Sim M. Beyond traditional body composition metrics: load-capacity indices emerge as predictors of cardiometabolic outcomes—a systematic review and meta-analysis. Adv Nutr. (2025) 16:100364. doi: 10.1016/j.advnut.2024.100364, PMID: 39756680 PMC11808523

[69] Cancello R Brunani A Brenna E Soranna D Bertoli S Zambon A. Phase angle (PhA) in overweight and obesity: evidence of applicability from diagnosis to weight changes in obesity treatment. Rev Endocr Metab Disord. (2022) 24:451–64. doi: 10.1007/S11154-022-09774-1, PMID: 36484943 PMC9735068

[70] Sagun G Oguz A Karagoz E Filizer AT Tamer G Mesci B. Application of alternative anthropometric measurements to predict metabolic syndrome. Clinics. (2014) 69:347–53. doi: 10.6061/CLINICS/2014(05)09, PMID: 24838901 PMC4012236

[71] Gallagher D Heymsfield SB Heo M Jebb SA Murgatroyd PR Sakamoto Y. Healthy percentage body fat ranges: an approach for developing guidelines based on body mass index. Am J Clin Nutr. (2000) 72:694–701. doi: 10.1093/ajcn/72.3.694, PMID: 10966886

[72] Cruz-Jentoft AJ Bahat G Bauer J Boirie Y Bruyère O Cederholm T. Sarcopenia: revised European consensus on definition and diagnosis. Age Ageing. (2019) 48:16–31. doi: 10.1093/AGEING/AFY169, PMID: 30312372 PMC6322506

[73] Leeflang MMG Rutjes AWS Reitsma JB Hooft L Bossuyt PMM. Variation of a test’s sensitivity and specificity with disease prevalence. Can Med Assoc J. (2013) 185:E537–44. doi: 10.1503/CMAJ.121286, PMID: 23798453 PMC3735771

[74] Murad MH Lin L Chu H Hasan B Alsibai RA Abbas AS. The association of sensitivity and specificity with disease prevalence: analysis of 6909 studies of diagnostic test accuracy. Can Med Assoc J. (2023) 195:E925–31. doi: 10.1503/CMAJ.221802/TAB-RELATED-CONTENT 37460126 PMC10356012

