# Supplementary material for: Body composition as a complementary tool for detection of metabolic syndrome 6 years postpartum: a St. Carlos Cohort follow-up
Source: Front Nutr. 2025 Oct 29;12:1689658. doi: 10.3389/fnut.2025.1689658 (PMC12614464; doi:10.3389/fnut.2025.1689658)
Supplement: Supplementary file 1 [file Table_1.DOCX]

| **SUPPLEMENTARY TABLE 1. Diet quality and physical activity at six years postpartum stratified by prior Gestational Diabetes Mellitus diagnosis (Mean ± SD)** | | | | | |
| --- | --- | --- | --- | --- | --- |
|  |  |  | NGT (n = 447) | GDM (n = 116) | P‒value |
| Total MEDAS |  | (score) | 8.52 ± 2.02 | 8.32 ± 1.99 | 0.319 |
|  |  |  |  |  |  |
| **Physical Activity** |  |  |  |  |  |
| Sitting time |  | (days) | 4.98 ± 3.11 | 5.03 ± 3.11 | 0.885 |
|  |  | (minutes) | 279.17 ± 194.03 | 278.8 ± 196 | 0.985 |
| Light |  | (days) | 5.62 ± 1.79 | 5.39 ± 1.87 | 0.229 |
|  |  | (minutes) | 62.67 ± 83.33 | 49.46 ± 72.65 | 0.119 |
|  |  | (MET/week) | 1085.83 ± 1337.62 | 765.86 ± 965.2 | 0.004 |
| Moderate |  | (days) | 2.78 ± 1.65 | 3.09 ± 2.17 | 0.436 |
|  |  | (minutes) | 65.47 ± 86.68 | 75.76 ± 84.02 | 0.533 |
|  |  | (MET/week) | 275.12 ± 927.36 | 240.69 ± 612 | 0.705 |
| Intense |  | (days) | 3.04 ± 1.8 | 3.86 ± 2.1 | 0.067 |
|  |  | (minutes) | 91.01 ± 80.77 | 104.05 ± 134.22 | 0.671 |
|  |  | (MET/week) | 570.92 ± 2126.14 | 583.1 ± 2690 | 0.959 |
| Total MET |  | (MET/week) | 1896.7 ± 2802.79 | 1589.65 ± 2919.6 | 0.298 |
| Physical Activity | Light | n (%) | 128 (27.9) | 42 (36.2) | 0.160 |
|  | Moderate | n (%) | 259 (56.4) | 61 (52.6) |  |
|  | Intense | n (%) | 72 (15.7) | 13 (11.2) |  |
| NTG, Normal Glucose Tolerance; GDM, Gestational Diabetes Mellitus; MEDAS, Mediterranean Diet Adherence Screener | | | | | |
