# Supplementary material for: Body composition as a complementary tool for detection of metabolic syndrome 6 years postpartum: a St. Carlos Cohort follow-up
Source: Front Nutr. 2025 Oct 29;12:1689658. doi: 10.3389/fnut.2025.1689658 (PMC12614464; doi:10.3389/fnut.2025.1689658)
Supplement: Supplementary file 2 [file Table_2.DOCX]

| **SUPPLEMENTARY TABLE 2. Biochemical parameters at six years postpartum stratified by prior Gestational Diabetes Mellitus diagnosis (Mean ± SD)** | | | | | | |
| --- | --- | --- | --- | --- | --- | --- |
|  | Normal ranges | | NGT (n = 480) | GDM (n = 124) | P‒value |  |
| Glucose | 74 ‒ 106 | mg/dL | 91.45 ± 6.69 | 96.98 ± 8.94 | 0.001 |  |
| HbA1c | 4 ‒ 5.7 | % | 5.31 ± 0.29 | 5.43 ± 0.34 | 0.001 |  |
| Insulin | 1.9 – 23 | µUI/mL | 6.75 ± 5.15 | 8.13 ± 5.81 | 0.012 |  |
| HOMA | 0.1 – 3 |  | 1.55 ± 1.25 | 2.08 ± 1.53 | 0.001 |  |
|  |  |  |  |  |  |  |
| Total Cholesterol | 25 – 200 | mg/dL | 185.78 ± 30.15 | 194.31 ± 33.51 | 0.006 |  |
| HDL Cholesterol | > 50 | mg/dL | 62.24 ± 12.17 | 61.42 ± 11.7 | 0.501 |  |
| LDL Cholesterol | < 130 | mg/dL | 108.1 ± 25.3 | 113.7 ± 29.23 | 0.033 |  |
| Triglycerides | 9 – 150 | mg/dL | 77.47 ± 37.03 | 95.76 ± 56.49 | 0.001 |  |
| Apo B | 55 – 125 | mg/dL | 84.9 ± 18.23 | 92.29 ± 22.23 | 0.001 |  |
|  |  |  |  |  |  |  |
| AST | 3 – 35 | U/L | 20.35 ± 5.76 | 22.01 ± 10.68 | 0.098 |  |
| ALT | 3 – 35 | U/L | 17.19 ± 9.89 | 20.31 ± 18.79 | 0.075 |  |
| GGT | 1 – 39 | U/L | 18.97 ± 13.36 | 20.79 ± 14.33 | 0.184 |  |
| Alkaline phosphatase | 33 – 120 | U/L | 62.47 ± 19.73 | 64.8 ± 19.68 | 0.241 |  |
| TSH | 0.3 – 5.3 | µUI/mL | 1.96 ± 1.47 | 1.88 ± 1.03 | 0.558 |  |
| Free T4 | 5.8 – 16.4 | pg/mL | 8.61 ± 1.39 | 8.58 ± 1.76 | 0.832 |  |
| hsCRP | ≤ 5 | mg/L | 1.29 ± 3.06 | 1.09 ± 1.81 | 0.496 |  |
| NTG, Normal Glucose Tolerance; GDM, Gestational Diabetes Mellitus; HOMA, Homeostatic model assessment; Apo B, apolipoprotein B; AST, aspartate aminotransferase; ALT, alanine aminotransferase; GGT, gamma–glutamyl transferase; TSH, thyroid–stimulating hormone; FT4, Free thyroxine; hsCRP, high sensitivity C–reactive protein | | | | | | |
