# Supplementary material for: Body composition as a complementary tool for detection of metabolic syndrome 6 years postpartum: a St. Carlos Cohort follow-up
Source: Front Nutr. 2025 Oct 29;12:1689658. doi: 10.3389/fnut.2025.1689658 (PMC12614464; doi:10.3389/fnut.2025.1689658)
Supplement: Supplementary file 3 [file Table_3.DOCX]

| **SUPPLEMENTARY TABLE 3**. **Anthropometric and body composition parameters six years postpartum stratified by Metabolic Syndrome Criteria and Gestational Diabetes Mellitus (Mean ± SD)** | | | | | | | | | | | | | | | |  |
| --- | --- | --- | --- | --- | --- | --- | --- | --- | --- | --- | --- | --- | --- | --- | --- | --- |
|  |  | 0 – 2 MetS Criteria (n = 501) | | | 3 MetS Criteria (n = 59) | | | | 4 MetS Criteria (n = 30) | | | | 5 MetS Criteria (n = 14) | | |  |
|  |  | NGT (n = 410) | GDM (n = 91) | P | NGT (n = 43) | GDM (n = 16) | P | NGT (n = 9) | | DGM (n = 21) | P | NGT (n = 6) | | DGM (n = 8) | P | |
| Weight | (kg) | 62.44 ± 9.64 | 62.17 ± 9.3 | 0.804 | 76.8 ± 10.93 | 77.61 ± 11.48 | 0.804 | 81.23 ± 11.77 | | 82.01 ± 9.5 | 0.862 | 83.93 ± 11.32 | | 79.54 ± 11.71 | 0.495 | |
| WC | (cm) | 76.01 ± 7.74 | 76.31 ± 7.11 | 0.733 | 90.22 ±7.09 | 90.73 ± 10.19 | 0.856 | 97.38 ± 8.26 | | 94.32 ± 9.73 | 0.385 | 94.95 ± 4.35 | | 100.08 ± 9.67 | 0.212 | |
|  |  |  |  |  |  |  |  |  | |  |  |  | |  |  | |
| Fat Mass (FM) | (kg) | 21.56 ± 6.8 | 21.42 ± 6.01 | 0.864 | 32.34 ± 7.39 | 32.87 ± 9.23 | 0.820 | 37.02 ± 7.79 | | 34.52 ± 6.92 | 0.414 | 37.25 ± 7.83 | | 33.92 ± 7.74 | 0.443 | |
|  | (%) | 33.8 ± 6.31 | 34.02 ± 5.65 | 0.753 | 41.69 ± 4.59 | 40.97 ± 7.5 | 0.656 | 45.44 ± 4.59 | | 41.75 ± 4.41 | 0.052 | 44.02 ± 3.82 | | 42.4 ± 5.81 | 0.566 | |
| FMI | (kg/m^2^) | 8.19 ± 2.59 | 8.16 ± 2.3 | 0.925 | 12.25 ± 2.68 | 12.67 ± 3.55 | 0.629 | 14.54 ± 2.80 | | 13.56 ± 2.90 | 0.399 | 15.04 ± 3.62 | | 13.72 ± 2.88 | 0.462 | |
| Visceral fat | (L) | 1 ± 0.56 | 1.01 ± 0.51 | 0.845 | 1.77 ± 0.62 | 1.82 ± 0.78 | 0.791 | 2.3 ± 0.62 | | 2.17 ± 0.79 | 0.634 | 2.3 ± 0.68 | | 2.17 ± 0.51 | 0.707 | |
| FFM | (kg) | 40.93 ± 4.2 | 40.87 ± 4.55 | 0.903 | 43.87 ± 4.03 | 44.97 ± 3.84 | 0.351 | 43.93 ± 5.4 | | 47.56 ± 3.73 | 0.078 | 46.67 ± 3.84 | | 43.23 ± 3.08 | 0.099 | |
| FFM Index | (kg/m^2^) | 15.68 ± 1.3 | 15.68 ± 1.35 | 0.976 | 17.15 ± 1.5 | 17.63 ± 1.52 | 0.277 | 17.56 ± 1.23 | | 18.47 ± 1.27 | 0.081 | 18.8 ± 1.08 | | 17.33 ± 2.89 | 0.307 | |
| FM/FFM |  | 0.52 ± 0.15 | 0.52 ± 0.13 | 0.965 | 0.72 ± 0.14 | 0.73 ± 0.17 | 0.910 | 0.84 ± 0.16 | | 0.72 ± 0.13 | 0.055 | 0.79 ± 0.12 | | 0.77 ± 0.19 | 0.845 | |
| SMM | (kg) | 17.8 ± 1.95 | 17.77 ± 2.43 | 0.888 | 18.97 ± 2.31 | 18.99 ± 1.6 | 0.974 | 18.94 ± 2.37 | | 19.84 ± 2.33 | 0.346 | 20.44 ± 1.21 | | 18.98 ± 2.98 | 0.282 | |
| SMMI | (kg/m^2^) | 6.74 ± 0.62 | 6.75 ± 0.71 | 0.903 | 7.18 ± 0.75 | 7.34 ± 0.75 | 0.482 | 7.34 ± 0.8 | | 7.77 ± 0.79 | 0.187 | 8.23 ± 0.75 | | 7.7 ± 1.05 | 0.314 | |
| SMM/Weight |  | 0.29 ± 0.04 | 0.29 ± 0.03 | 0.815 | 0.25 ± 0.02 | 0.25 ± 0.03 | 0.910 | 0.23 ± 0.02 | | 0.24 ± 0.03 | 0.230 | 0.25 ± 0.03 | | 0.24 ± 0.03 | 0.704 | |
| ASM | (kg) | 14.62 ± 1.61 | 14.62 ± 1.75 | 0.988 | 16.52 ± 2 | 16.64 ± 1.72 | 0.833 | 17 ± 1.97 | | 17.41 ± 1.65 | 0.591 | 17.72 ± 1.13 | | 17.02 ± 2.34 | 0.518 | |
| ASMI | (kg/m^2^) | 5.54 ± 0.53 | 5.56 ± 0.52 | 0.731 | 6.25 ± 0.6 | 6.42 ± 0.67 | 0.359 | 6.59 ± 0.68 | | 6.82 ± 0.58 | 0.378 | 7.13 ± 0.65 | | 6.9 ± 0.72 | 0.546 | |
| ASM/BMI |  | 0.62 ± 0.07 | 0.62 ± 0.07 | 0.763 | 0.56 ± 0.06 | 0.55 ± 0.05 | 0.495 | 0.52 ± 0.06 | | 0.55 ± 0.06 | 0.243 | 0.52 ± 0.04 | | 0.51 ± 0.06 | 0.829 | |
| TBW | (L) | 30.44 ± 3.26 | 30.28 ± 3.69 | 0.675 | 32.8 ± 3.01 | 33.59 ± 3.05 | 0.373 | 32.87 ± 3.82 | | 35.72 ± 2.85 | 0.054 | 35.15 ± 3.02 | | 32.34 ± 2.16 | 0.077 | |
|  | (%) | 48.86 ± 4.45 | 48.82 ± 3.87 | 0.937 | 43.33 ± 3.38 | 43.49 ± 4.57 | 0.887 | 40.77 ± 3.27 | | 43.52 ± 3.35 | 0.044 | 41.9 ± 2.69 | | 42.76 ± 4.15 | 0.666 | |
| ECW | (L) | 13.39 ± 1.49 | 13.46 ± 1.66 | 0.730 | 14.6 ± 1.36 | 14.64 ± 1.14 | 0.936 | 14.81 ± 1.2 | | 15.6 ± 1.81 | 0.328 | 15.43 ± 0.91 | | 14.27 ± 1.17 | 0.178 | |
| ECW/TBW |  | 0.45 ± 0.02 | 0.45 ± 0.03 | 0.185 | 0.44 ± 0.02 | 0.45 ± 0.02 | 0.425 | 0.45 ± 0.01 | | 0.44 ± 0.02 | 0.296 | 0.45 ± 0.01 | | 0.44 ± 0.02 | 0.378 | |
| PhA | ( º ) | 5 ± 0.47 | 5.01 ± 0.47 | 0.889 | 5.15 ± 0.51 | 5.28 ± 0.57 | 0.430 | 5.32 ± 0.53 | | 5.53 ± 0.34 | 0.275 | 5.45 ± 0.7 | | 5.66 ± 0.42 | 0.522 | |
| PhA/BMI |  | 0.21 ± 0.03 | 0.21 ± 0.03 | 0.942 | 0.18 ± 0.03 | 0.18 ±0.03 | 0.893 | 0.17 ± 0.02 | | 0.18 ± 0.22 | 0.236 | 0.16 ± 0.03 | | 0.17 ± 0.03 | 0.514 | |
| Resistance | (ohm) | 688.41 ± 68.95 | 684.35 ± 75.75 | 0.620 | 639.29 ± 72.43 | 618.41 ± 61.07 | 0.309 | 626.62 ± 66.49 | | 589.19 ± 57.8 | 0.154 | 548.88 ± 60.64 | | 593.05 ± 81.41 | 0.287 | |
| Reactance | (ohm) | 60.08 ± 6.96 | 60.49 ± 7.74 | 0.618 | 57.62 ± 7.77 | 56.87 ± 6.87 | 0.736 | 58.06 ± 5.34 | | 56.94 ± 4.82 | 0.593 | 52.18 ± 8.89 | | 59.61 ± 7.21 | 0.109 | |
| MetS, Metabolic Syndrome; NTG, Normal Glucose Tolerance; DGM, Gestational Diabetes Mellitus; FM, Fat Mass; FFM, Fat Free Mass; WC, Waist Circumference; SMM, Skeletal muscle mass; SMMI, Skeletal muscle mass index; SMI, Skeletal muscle index; ASM, Appendicular skeletal muscle mass; ASMI, Appendicular skeletal muscle mass index; TBW, Total Body Water; ECW, extracellular water; TBW, total body water. | | | | | | | | | | | | | | | |  |
